# Efficacy of wrist robot-aided orthopedic rehabilitation: a randomized controlled trial

**DOI:** 10.1186/s12984-021-00925-0

**Published:** 2021-08-31

**Authors:** Giulia Aurora Albanese, Elisa Taglione, Cecilia Gasparini, Sara Grandi, Foebe Pettinelli, Claudio Sardelli, Paolo Catitti, Giulio Sandini, Lorenzo Masia, Jacopo Zenzeri

**Affiliations:** 1 Robotics, Brain and Cognitive Sciences (RBCS) Unit, Istituto Italiano di Tecnologia, Genoa, Italy; 2 Department of Informatics, Bioengineering, Robotics and Systems Engineering (DIBRIS), University of Genoa, Genoa, Italy; 3 National Institute for Insurance against Accidents at Work (INAIL), Motor Rehabilitation Center, Volterra, Italy; 4 Institut für Technische Informatik (ZITI), Heidelberg University, Heidelberg, Germany

**Keywords:** Robotic rehabilitation, Robotic assessment, Wrist injury, Orthopedic

## Abstract

**Background:**

In recent years, many studies focused on the use of robotic devices for both the assessment and the neuro-motor reeducation of upper limb in subjects after stroke, spinal cord injuries or affected by neurological disorders. Contrarily, it is still hard to find examples of robot-aided assessment and rehabilitation after traumatic injuries in the orthopedic field. However, those benefits related to the use of robotic devices are expected also in orthopedic functional reeducation.

**Methods:**

After a wrist injury occurred at their workplace, wrist functionality of twenty-three subjects was evaluated through a robot-based assessment and clinical measures (Patient Rated Wrist Evaluation, Jebsen-Taylor and Jamar Test), before and after a 3-week long rehabilitative treatment. Subjects were randomized in two groups: while the control group (n = 13) underwent a traditional rehabilitative protocol, the experimental group (n = 10) was treated replacing traditional exercises with robot-aided ones.

**Results:**

Functionality, assessed through the function subscale of PRWE scale, improved in both groups (experimental p = 0.016; control p < 0.001) and was comparable between groups, both pre (U = 45.5, p = 0.355) and post (U = 47, p = 0.597) treatment. Additionally, even though groups’ performance during the robotic assessment was comparable before the treatment (U = 36, p = 0.077), after rehabilitation the experimental group presented better results than the control one (U = 26, p = 0.015).

**Conclusions:**

This work can be considered a starting point for introducing the use of robotic devices in the orthopedic field. The robot-aided rehabilitative treatment was effective and comparable to the traditional one. Preserving efficacy and safety conditions, a systematic use of these devices could lead to decrease human therapists’ effort, increase repeatability and accuracy of assessments, and promote subject’s engagement and voluntary participation. *Trial Registration* ClinicalTrial.gov ID: NCT04739644. Registered on February 4, 2021—Retrospectively registered, https://www.clinicaltrials.gov/ct2/show/study/NCT04739644.

## Background

Wrist traumatic injuries usually lead to hand motor control deficits and loss of functionality, as direct consequences of both the lesion itself and the following immobilization period. Actually, occurrence of tissue rigidity, lack of stretch, muscle strength reduction, pain and edema could bring to a limited Range Of Motion (ROM) along some directions of movement [1]. Moreover, long periods of immobilization could take to proprioceptive deficits, preventing post-traumatic subjects to have a proper control of movements and worsening their performance during fine manipulation tasks [2–4]. Wrist injuries could differ in terms of severity and site: lesions may involve flexor or extensor tendons, complexes made of fibrocartilage, ligaments, bones or more than one of these tissues [1]. Each tissue presents a different vascularization and consequently a different healing time [5], while the site of the lesion has a direct influence on the resulting functional impairment, such as the movement direction more compromised [6]. For these reasons and because of age differences, both orthopedic treatments and following rehabilitative approaches could differ between subjects [7–10]. In terms of orthopedic treatments, we can identify conservative and surgery interventions: while the former implies the application of a cast or a splint, the latter allows to shorter periods of immobilization, decreasing all the related problems, such as rigidity, loss of strength and altered proprioception [11]. Generally, the choice of a surgical or non-surgical approach seems to have an influence on the grip force, reduced for non-surgical cases, but it has been demonstrated the absence of significant differences in terms of motor and sensory impairments [3]. Removed the cast or splint, rehabilitation programs should begin as soon as possible. Rehabilitative interventions have the goal of restoring functional abilities and subject’s self-sufficiency: therapists tailor these protocols to meet the need presented by individual patients, in terms of duration, intensity and exercises. A large variety of exercises is conventionally used in clinics: active and passive joint mobilizations, continuous motion and strengthening exercises supervised and assisted by physical therapists, supportive splints, physical methods of pain management, but also occupational therapy programs and self-administered exercises [7–10]. However, some standardized protocols of ordinary physical interventions are usually applied [10]: treated the presence of pain and edema, initially only active exercises to stretch soft tissues and improve the range of motion are allowed; passive mobilizations, strengthening and proprioceptive exercises are gradually introduced in the following weeks [7, 8]. Particularly, both active and passive exercises for increasing the ROM have a relevant level of evidence supporting their usage [12].

Nowadays, physical therapists work with traditional devices for therapeutic exercise, common in every rehabilitative center, or employ their own strength for manual mobilizations. In contrast to what has been happening in the neuro-rehabilitative field [13–15], in the orthopedic one examples of robot-aided assessment and rehabilitation of wrist injuries are hard to find [16, 17]. However, analogously to what has been observed in the neuro-motor reeducation of the upper limb in subjects after stroke or spinal cord injuries [18–21], advantages are expected from the use of these devices for the functional reeducation after wrist traumatic injuries. Robot-assisted therapy meets the need of orthopedic patients for a personalized protocol and a maximized training effect, allowing levels of assistance or resistance tailored on the real-time performance. Actually, high-resolution recording of spatial and temporal data allows to compute novel performance indicators [6, 22, 23] and document constantly progresses related to therapy, assessed under repeatable and safe conditions. For these reasons, the use of a robotic system with post-traumatic subjects could decrease human therapists’ effort and increase the efficiency in terms of both treatment duration and final reached functionality. Finally, the possibility to couple therapy with a virtual reality environment could be useful to increase patient’s participation, engagement and motivation, demonstrated to be related to the treatment success or failure [17, 24–26].

For all the above-mentioned reasons, this randomized clinical trial aimed to address three main questions about the use of a robotic device for the rehabilitative training of subjects presenting wrist injuries: (1) whether a robot-based rehabilitative approach is effective on wrist functionality; (2) whether the effects of robot-based rehabilitation are different from those achieved through a conventional therapy; (3) which is the acceptability of this novel approach perceived by patients. In the present work, we introduce the structure of the clinical trial and the results obtained from robotic evaluations, clinical measures and asked approval rating.

## Methods

### Experimental setup

The study was carried out at the INAIL Motor Rehabilitation Center (Volterra, Italy) and involved the employment of the WRISTBOT, a robotic device developed at the Istituto Italiano di Tecnologia (Genoa, Italy) [27, 28]. This robot was designed for and is currently employed in motor control and rehabilitation studies of the human wrist [23, 29–31]. The WRISTBOT is a fully backdrivable manipulandum that allows for movements along its 3 Degrees of Freedom (DoFs) in a human-like Range Of Motion (ROM) of the wrist: ± 62° flexion/extension (FE), − 40°/ + 45° in radial/ulnar deviation (RUD), and ± 60° pronation/supination (PS). In addition, the robot permits motions along planes that involve combined multi-DoFs movements. Mechanically, the robot was developed to have low values of inertia, emulating the fluency of natural movements. Each DOF is measured by high resolution incremental encoders and actuated by one brushless motor or two in case of the RUD planes, providing both gravity compensation and continuous torque values necessary to manipulate the human wrist joints. The torque ranges at the different wrist joints are 1.53 Nm on FE, 1.63 Nm on RUD and 2.77 Nm on PS. Depending on the torques exerted, the device can be used in either active or assistive/passive modality. While the active modality requires only subject’s active muscle work, the assistive/passive one was implemented using an impedance control scheme, based on the real-time relative position between the target and the end-effector, with a 1 kHz sampling frequency. The system is integrated with a Virtual Reality environment (VR), useful to provide a visual feedback to the user while he/she is requested to complete the tasks.

### Subjects and experimental protocol

The design of this study was an interventional, parallel, and randomized clinical trial on a consecutive convenience sample of 27 subjects. Using a computer, a therapist randomly assigned subjects to each group, whose main characteristics are reported in Table 1. Twenty-three subjects completed the entire protocol, because of three dropouts in the experimental group (n = 10) and one among control subjects (n = 13). The dropouts were due to the impossibility to have a constant routine of at least 4 training sessions performed each week. In particular, some inclusion criteria have to be fulfilled to participate the study: adults of both sexes, aged between 18 and 65 years, presenting functional and spatial limitations of the wrist joint, following an injury occurred at their workplace. Participants’ injuries included scapholunate ligament injuries, distal radius/ ulnar fractures, carpal bones fractures or dislocations, triangular fibrocartilage complex (TFCC) injuries. In details, subjects had to be in the post-immobilization phase and the temporal distance from the acute event did not have to exceed 6 months. Exclusion criteria were non-compliance with study requirements, pregnancy or breast feeding, prior history of malignancy, contraindications to wrist passive movements, acute inflammatory arthritis of the wrist, open skin at the level of the patient-device interface. The research was performed in accordance with the Declaration of Helsinki and approved by the local ethics committee (protocol number 76, code CRMINAIL03). An informed consent was signed to participate to the study.

**Table 1:** Subjects’ characteristics and distribution in the experimental and control group

	Experimental (n = 10)	Control (n = 13)
Sex (male/female)	5/5	9/4
Age (mean ± std)	48.7 ± 11.8 years	50.9 ± 9.1 years
Right-handed	9	11
Dominant side injured	6	8
Lesion: Fractures/dislocations	5	8
Ligament injuries	3	5
Both	1	–
Others (TFCC lesion)	1	–
Orthopedic treatment (surgery intervention/conservative treatment)	8/2	10/3
Temporal distance between the acute event and the 1st evaluation (days, mean ± SD)	98.5 ± 44.0	101.60 ± 43.3

**Fig. 1:**
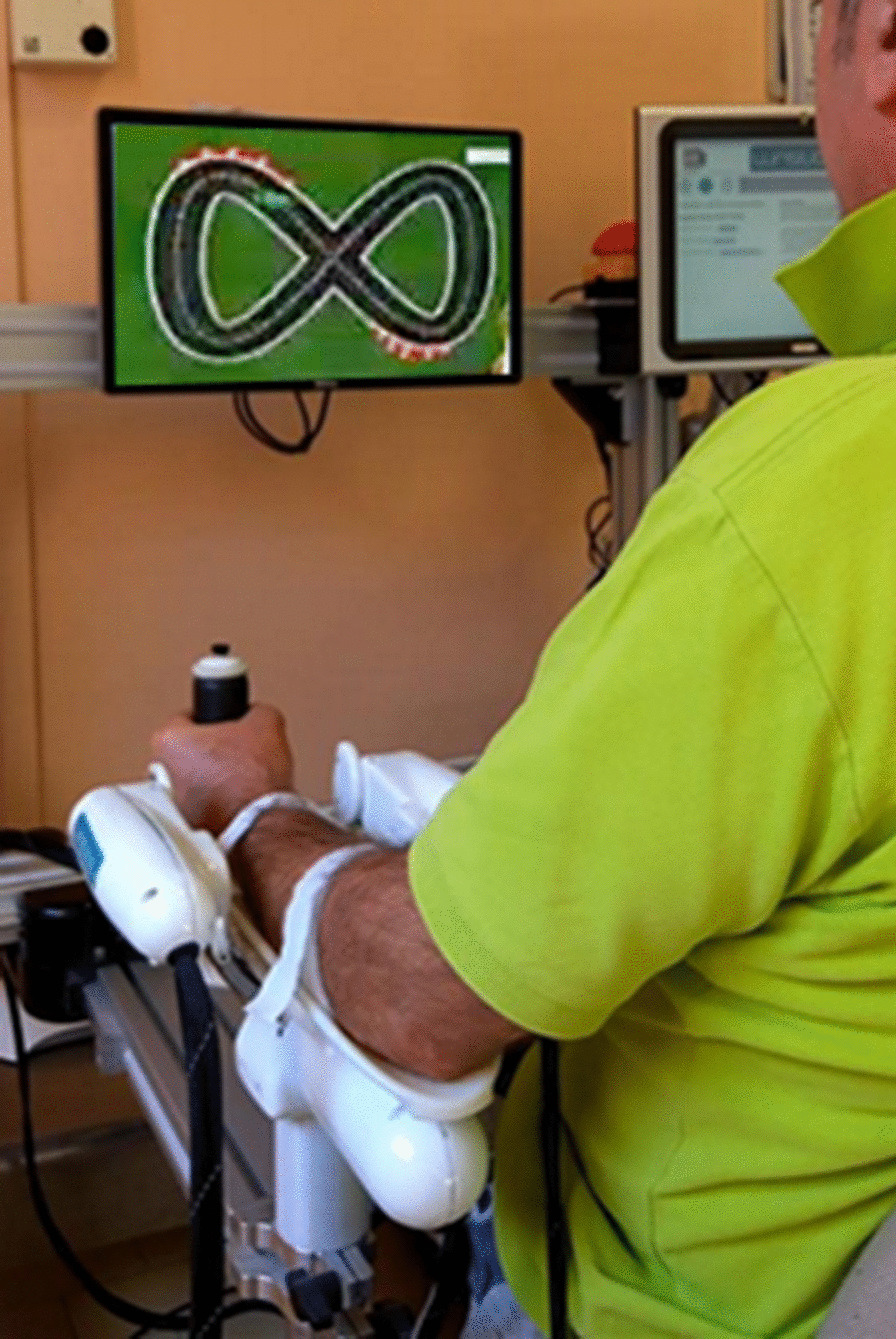
The experimental setup. Subjects’ placement and virtual reality during an illustrative example of a tracking task

During robot-aided sessions, subjects sat on a chair in front of a screen, holding the handle of the robot, with their forearm strapped to the robot support to assure a correct alignment between the axes of the mechanical structure and the wrist’s rotational ones (Fig. 1).

**Fig. 2:**
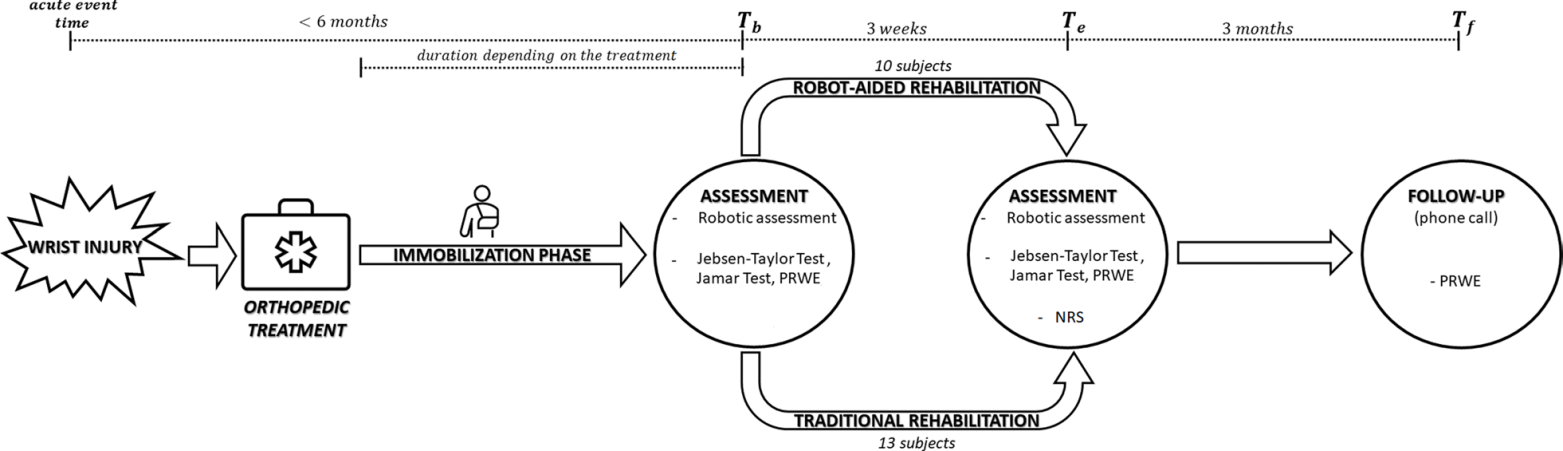
The experimental protocol

The protocol included both assessment and rehabilitative sessions (Fig. 2). All subjects performed the same sessions of assessment, which included two evaluations through the robotic system and clinical measures at the beginning (T_b_) and at the end (T_e_) of the rehabilitative training, and a follow up through phone call, three months after the end of the treatment (T_f_).

Three clinical measures were assessed at T_b_ and T_e_ as primary outcomes:

1. Jamar Test [32]: using a hand dynamometer, subjects performed three trials to evaluate the mean static palmar force exerted in kg.
2. Jebsen Taylor Hand Function Test (JTHFT) [33, 34]: consisting of six items, its aim was to evaluate dexterity in terms of fine motor skills, weighted functional tasks and non-weighted functional tasks. Each item is scored according to the time taken to complete the task.
3. Patient Rated Wrist/Hand Evaluation (PRWE) [35–37]: a questionnaire composed of a pain (PRWE-P) and a function (PRWE-F) subscale. Each subsection has a maximum score of 50 and a minimum of 0, where less score points out a better performance. PRWE was the only assessment involved also in the follow-up call (T_f_).

On the same days (T_b_ and T_e_), subjects performed also the robot-aided evaluation. Each robotic assessment lasted around 45 min and followed a 15-min session to familiarize with the device, assuring all subjects to be similarly acquainted with the device. Rehabilitative training was not performed during assessment days.

Robot-aided evaluative session included five exercises, useful to assess robotic primary outcomes as ROM, exerted forces, dexterity, and wrist position sense acuity:

1. *Passive ROM* Starting from the neutral position (0° along each DoF), the device moved the wrist of the subject along different directions until subject’s maximum tolerance, notified by himself/herself pushing a button with the not injured hand. Subjects let the robot moving their wrist passively, without opposing or facilitating the motion. Target directions were 8 equally distributed in the FE/RUD space [radial deviation (RD), ulnar deviation (UD), flexion F, extension (E), ulnar flexion (UF), ulnar extension (UE), radial flexion (RF) and radial extension (RE)], and 2 along PS [pronation (P), supination (S)]. Passive ROM assessment was necessary to choose appropriate target positions and distances in some of the following exercises (Target Tracking and Joint Position Matching), suitable for the severity of the injury of the single subject and his/her level of healing. Outcome measures consisted in the maximum ROM in degrees achieved along each direction.
2. *Active ROM* From the initial neutral position, subjects moved actively the device as far as they could, along the same directions of the previous exercise. Any assistive force was applied, but the weight of the device was compensated during active motions. The outcome measure was the maximum active ROM in degrees achieved along each direction.
3. *Isometric Force* While the device kept subjects on the wrist neutral position, they were requested to perform a maximal contraction toward different directions. While subjects pushed towards each target direction, the device resisted to the imposed force, such that no motion was performed. The outcome measure was maximal peak force in Newton measured along each direction (same directions as in ROM assessment).
4. *Target Tracking* Subjects had to follow a target moving on a first order Lissajous trajectory, showed on the screen two-dimensional space. Subjects performed two laps, actively moving in two different directions of rotation (counter and clockwise) across the space described by combinations of FE and RUD motions. The size of the figure was determined by the 75% of the smallest assessed ROM among UD, RD, F and E directions. The resulting outcome measure was the mean figural error in degrees, i.e. the average angular distance between target and end-effector trajectory in each sampled point [38].
5. *Joint Position Matching* While the subject was blindfolded, the device moves his/her wrist in a defined direction, until the 75% of the subject’s ROM along that direction. After 3 s, the wrist was passively brought back to the neutral position. Then, maintaining the blindfolded condition, the subject was asked to reproduce the joint configuration previously assumed passively. Target directions corresponded to the same directions along which the ROM has been assessed. Performance was measured in terms of matching error, i.e., the Euclidean distance between target and matched points. Matching error was measured in degrees, since each-DoF rotational measurements were considered as single coordinates to compute distances [39].

In order to avoid inflammatory issues, each exercise was repeated once during each evaluation.

Finally, subjects were asked to indicate their approval rating through a Numerical Rating Scale (NRS) [40, 41]: subjects’ level of satisfaction about treatment was asked as secondary outcome at the end of the rehabilitative treatment (T_e_).

Concerning the rehabilitative training, the protocol included a three-week long rehabilitation, during which subjects performed 4/5 sessions per week, since the first and the last day were exclusively dedicated to the assessment. Each session lasted around 90 min and its structure was individually chosen by the medical specialist, according to the severity of the injury of each single subject. The control group was treated with conventional rehabilitative treatments, supervised and assisted by a therapist, using traditional tools and devices necessary for therapeutic exercises.

The experimental group underwent a comparable rehabilitative treatment, decided and supervised by the medical specialist, except for the replacement of some traditional manual exercises with robot-based ones. In details, replaced training exercises aimed to improve the ROM, muscle strength and dexterity (Table 2).

**Table 2:** Exercises included in the traditional and in the robotic rehabilitative training. In the experimental group, exercises in the “Traditional training” column have been replaced with those in the corresponding row of the “Robotic training” column

Traditional training			Robotic training		
Headline	Description	Duration (min)	Headline	Description	Duration (min)
Passive mobilization	Subsequent series of movements along different directions, supported by the physiotherapist	15	Passive mobilization	Subsequent series of passive mobilizations, performed applying constant forces to move the hand across the 3-DoFs space	15
Active and assisted mobilization	Subsequent series of movements along different directions, initially assisted by the physiotherapist	15	Active and assisted mobilization	Subsequent series tracing movements along linear trajectories, with a robotic assistance proportional to the distance from the final point	15
Exercise with elastic bands or weights	Subsequent series of contraction along different movement planes, using an elastic band or with gradually increasing weights	20	Reaching with elastic resistance	Subsequent series of reaching across the FE-RUD space, in presence of constant or distance-dependent resistive forces	20
Exercise of manipulation and dexterity	Simulation of daily life activities, supervised by the physiotherapist	40	Tracking	3-DoFs tracking of a moving target in a viscous field	40

Before each robotic training session, the passive ROM of participants was assessed through the dedicated exercise, because of safety reasons and in order to tailor target positions in the immediately following rehabilitative session. To avoid a learning effect related to a more prolonged use of the device in the experimental group, therapeutic robot-aided exercises were designed with the aim to be deeply different from those used in the sessions of assessment.

### Data processing

Given each-DoF encoder recordings during robotic assessments, data of joint rotations were re-sampled at a uniform 100 Hz sample rate by linear interpolation and filtered with a sixth order Savitzky-Golay low-pass filter (8 Hz cut-off frequency). While these represented performed angular displacements, the amount of current delivered by motors was used to estimate the forces exerted to assist or resist to subjects’ motion.

Computed the above-mentioned outcome measures for each exercise (see “Subjects and experimental protocol”), we obtained and statistically tested single-subject performance along each direction involved in the five exercises included in the assessment. Data smaller than 1.5 of the interquartile range (IQR) from the first quartiles or bigger than 1.5 IQR from the third one were considered outliers.

Additionally, we chose to inspect the whole performance of single exercises. The issue we had to face here was that, even in healthy populations, motor or perceptive performance along different directions is not comparable. For these reasons, we normalized single-direction performance of each subject respect to our sample performance along that direction. In details, for each subject and exercise, each single-direction outcome measure *x* was normalized as shown in the following equation:

1 $$x_{norm} = \frac{{x_{best} - x}}{{x_{best} - x_{worst} }}$$

where *x*_*best*_ and *x*_*worst*_ represent respectively the best and worst performance found along that specific direction of the considered exercise, among all the subjects and evaluations. In particularly, while in case of ROM or force exerted a better performance is reflected in higher output values, in all the other exercises higher outcome measures represented worse performances, since in those cases our metric was an error. Since our sample presented a wide range of injuries and different functional issues, some subjects showed values comparable to not-injured subjects [22, 39], already at the first evaluation. Therefore, this normalization scaled our measures respect to a range from a not injured to an injured wrist performance, along each specific direction of each exercise. Computed values resulted to be in a [0, 1] range, where lower values represented a better performance.

Next, for each exercise, we computed the median value within normalized single-directions, getting a single outcome measure in the [0, 1] range for each exercise (${outcome}_{exercise}$), whose meaning was how far from a healthy-like wrist functionality goal the subject performed.

Finally, given one outcome indicator for each out of the five exercises in each session of assessment (${outcome}_{\mathrm{exercise}}$), we compute an equally-weighted linear combination of them (Eq. 2):

2 $$Robotic \, Assessment \, Index = \mathop \sum \limits_{exercise = 1}^{5} outcome_{exercise}$$

Briefly, for each subject, this score (*Robotic Assessment Index*) reflected the global performance during single sessions of robotic assessment, with lower values suggesting a better performance.

### Statistical analysis

Statistical analysis involved single-direction measures, single-exercise outcomes, the *Robotic Assessment Index*, clinical measures and NRS results. Normality of data was inspected through Shapiro–Wilk Tests: these revealed presences of non-normally distributed data, which led to choose non-parametric tests for the statistical analysis. Mann–Whitney U tests have been performed to statistically analyze between-group performance at each session of assessment (T_b_, T_e_ and T_f_). Dependent non–parametric Wilcoxon Matched Pairs tests have been used to compare outcome metrics at T_b_ with those at T_e_, within each group (experimental/ control). Conversely, since PRWE was the only metric assessed three times (T_b_, T_e_ and T_f_), PRWE of each group was statistically analyzed through Friedman tests. Whether a main effect of measurement time was found, post-hoc pairwise comparisons were conducted through Durbin-Conover tests. Multiple comparisons were adjusted with a Bonferroni correction. Differences were considered significant when p < 0.050. Jamovi Statistical Data Analysis tool (JSDA, version 1.2.27) was used to conduct statistical analysis.

## Results

### Clinical measures

**Fig. 3:**
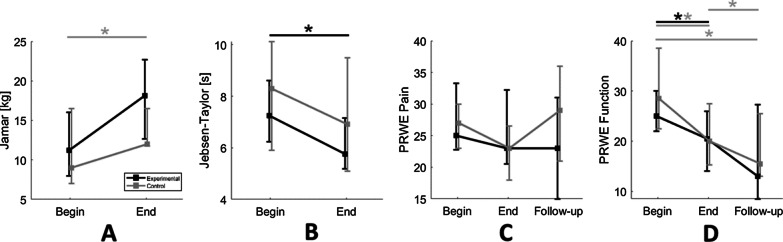
Median values and IQR of clinical tests scores. Panels presented results for Jamar Test (**A**), Jebsen-Taylor test (**B**), PRWE subscale pain (**C**) and PRWE subscale function (**D**). Grey and black lines stay for control and experimental group, respectively. Significant results in Wilcoxon Matched Pairs tests (p < 0.05) are identified by a “*”, black or grey depending on the tested group

Figure 3 show what has been found from clinical tests conducted by therapists, on the same days during which the robotic assessments were performed. The Jamar Test, assessing the grip force, showed values at T_e_ greater than T_b_: even though groups did not present significant differences both pre (U = 57, p = 0.642) and post rehabilitation (U = 45, p = 0.222), the grip force increase from T_b_ to T_e_ was significant in the control group (experimental: W = 6, p = 0.058; control: W = 12, p = 0.021). Conversely, in the Jebsen-Taylor test, only the experimental group presented a significant improvement (experimental: W = 52, p = 0.010; control: W = 43, p = 0.126), despite initial and final scores comparable between groups (T_b_: U = 58.5, p = 0.710; T_e_: U = 42, p = 0.387). Friedman tests on PRWE revealed that rehabilitation had effects mainly on the function subscale: pain subscale results presented neither significant difference between groups at all evaluations (T_b_: U = 49.5, p = 0.774; T_e_: U = 46, p = 0.594; T_f_: U = 26, p = 0.372), nor single-group improvements, represented by absence of significantly different scores among evaluations (experimental: χ^2^ = 2.82, p = 0.244; control: χ^2^ = 2.17, p = 0.338). On the other hand, both rehabilitative treatments had a significant effect on the function subscale (experimental: χ^2^ = 2.84, p = 0.016; control: χ^2^ = 16.8, p < 0.001). Pairwise comparison showed that, while the control group kept on improving also after 3 months (T_b_ vs T_e_: D–C = 5.49, p < 0.001; T_b_ vs T_f_: D–C = 7.82, p < 0.001; T_e_ vs T_f_: D–C = 2.32, p = 0.012), the experimental group did not reveal any significant change from the end of the treatment to the follow-up call (T_b_ vs T_e_: D–C = 2.65, p = 0.036; T_b_ vs T_f_: D-C = 4.08, p = 0.088; T_e_ vs T_f_: D–C = 1.43, p = 1.000). Nevertheless, independent Mann–Whitney U tests revealed that group scores were anyhow comparable at each evaluation (T_b_: U = 45.5, p = 0.355; T_e_: U = 47, p = 0.597; T_f_: U = 37, p = 0.703).

### Robotic Assessment Index

**Fig. 4:**
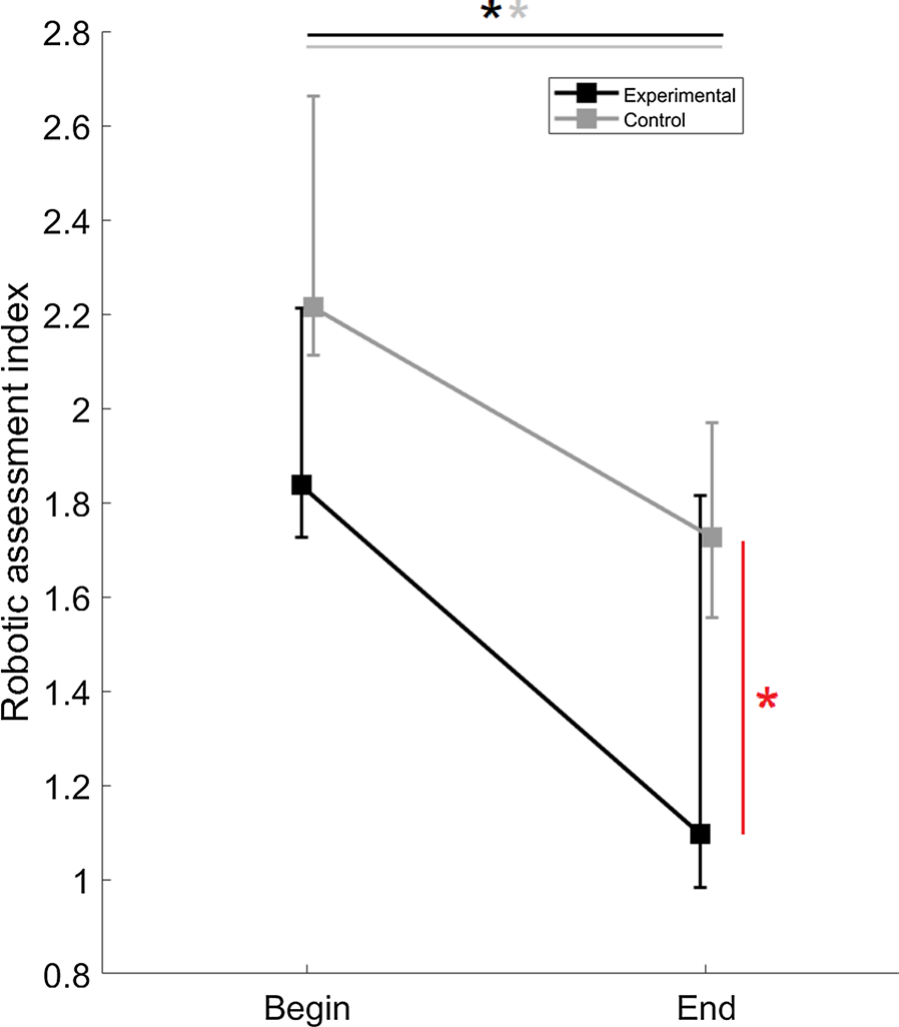
Median values and IQR of* Robotic Assessment Index*. Grey and black lines stay for control and experimental group, respectively. Significant results in Wilcoxon Matched Pairs tests are identified by a “*”, black for the experimental group and grey for the control one. Red “*” identified significant differences found after independent Mann–Whitney U tests

Figure 4 shows the scores of each group during single sessions of robotic assessment, computed considering the performance of all the five exercises. Wilcoxon Matched Pairs tests showed that both robotic and traditional training led to an improved performance in the robot-aided assessment after rehabilitation (experimental: W = 54, p = 0.004; control: W = 87, p = 0.002). However, although groups were comparable before the rehabilitative treatment (U = 36, p = 0.077), Mann–Whitney U tests revealed that the experimental group presented a significantly lower *Robotic Assessment Index* after the rehabilitative treatment (U = 26, p = 0.015), pointing out a final performance better than the control group. Given this global idea, we moved to analyze single exercises of assessment, with the aim of understanding the weight each component had on the whole assessment.

### Range of motion

As described by median values and IQRs in Table 3, groups presented comparable values of both active and passive ROM at T_b_ (*outcome*_*passiveROM*_: U = 53, p = 0.483; *outcome*_*activeROM*_: U = 64, p = 0.976). However, after three weeks of rehabilitative exercises, the experimental group presented a ROM greater than the control group (*outcome*_*passiveROM*_: U = 15, p = 0.001; *outcome*_*activeROM*_: U = 22, p = 0.006) and showed significant different values respect to pre-treatment ones (*outcome*_*passiveROM*_: W = 54, p = 0.004; *outcome*_*activeROM*_: W = 53, p = 0.006). Conversely, the control group presented a significant improvement from T_b_ to T_e_ only for active ROM (*outcome*_*passiveROM*_: W = 68, p = 0.127; *outcome*_*activeROM*_: W = 78, p = 0.021).

**Table 3:** Median normalized values (*outcome*_*exercise*_) and IQR of each exercise. For all the exercises, lower values stay for a better performance

Median values (IQR)	T_b_		T_e_	
	Experimental	Control	Experimental	Control
Passive ROM	0.108 (0.202–0.082)	0.161 (0.24–0.103)	0.0841 (0.0999–0.0501)	0.114 (0.241–0.105)
Active ROM	0.615 (0.631–0.496)	0.619 (0.722–0.475)	0.354 (0.414–0.171)	0.466 (0.521–0.438)
Isometric force	0.663 (0.809–0.576)	0.836 (0.877–0.771)	0.433 (0.556–0.309)	0.550 (0.759–0.483)
Target tracking	0.197 (0.288–0.124)	0.207 (0.369–0.132)	0.122 (0.209–0.089)	0.170 (0.279–0.149)
Joint position matching	0.298(0.389–0.185)	0. 352(0.424–0.288)	0. 285 (0.456–0.199)	0.318 (0.352–0.160)

**Fig. 5:**
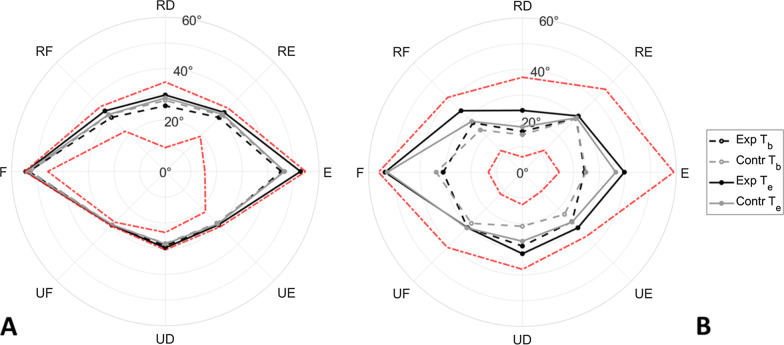
Median values of passive (**A**) and active (**B**) ROM. Black and grey lines stay for experimental and control group, respectively. Dotted and solid lines stay for T_b_ and T_e_ assessment, respectively. Red dotted lines represented data used to normalize each direction, i.e., the best and worst performance (x_best_ and x_worst_) found along that specific direction, among all the subjects and evaluations

**Fig. 6:**
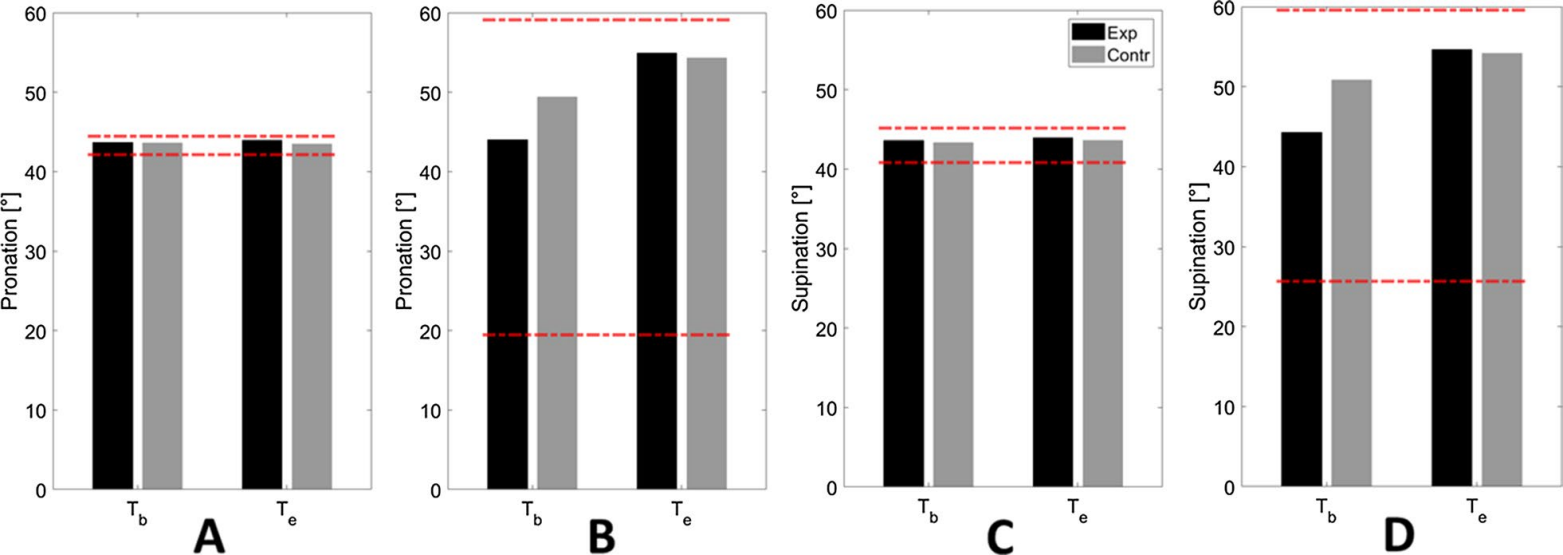
Median values of passive (**A** and **C**) and active (**B** and **D**) ROM for pronation (**A** and **B**) and supination movements (**C** and **D**). Black and grey stay for experimental and control group, respectively. Red dotted lines represented data used to normalize each direction, i.e., the best and worst performance (*x*_*best*_ and* x*_*worst*_) found along that specific direction, among all the subjects and evaluations

Detailed results related to single directions are reported in Table 4 and showed in Figs. 5, 6. Their trend and significance in both Mann–Whitney U and Wilcoxon Matched Pairs tests confirmed what has been found from exercise median normalized results (*outcome*_*passiveROM*_ / *outcome*_*activeROM*_): despite initial comparable values, in the second evaluation the experimental group showed a larger set of directions presenting significant ROM improvements, particularly in the passive assessment (Table 4).

**Table 4:** Statistical results for single directions

	RF		RD		E		UF		F		P		S		UE		RE		UD	
	Statistic	p-values	Statistic	p-values	Statistic	p-values	Statistic	p-values	Statistic	p-values	Statistic	p-values	Statistic	p-values	Statistic	p-values	Statistic	p-values	Statistic	p-values
Passive ROM																				
Wilcoxon Matched Pairs tests (experimental vs control)																				
Tb	59	0.974	57	0.948	63	0.927	37	0.353	45	0.851	51	0.809	29	0.131	37	0.353	50	0.601	31	0.099
Te	26	**0.049**	34	0.169	24	**0.021**	15	**0.024**	35	0.280	32	**0.042**	34	0.152	11	0.004*	31	0.111	18	0.004*
Mann–Whitney U (Tb vs Te)																				
Experimental	0	0.004*	0	**0.008**	0	0.004*	3	**0.020**	2	**0.023**	11	0.105	6	0.055	5	**0.020**	1	**0.016**	13	0.160
Control	14	0.102	12	**0.034**	29	0.273	9	0.469	11	0.203	32	0.966	9	0.129	1	0.063	17	0.092	16	0.275
Active ROM																				
Wilcoxon Matched Pairs tests (experimental vs control)																				
Tb	56	0.605	54	0.973	43	0.905	43	0.186	58	0.693	45	0.512	37	0.353	55	0.563	64	0.976	50	0.376
Te	20	**0.009**	34	0.110	48	0.512	40	0.699	65	1	40	0.503	36	0.384	18	**0.030**	11	0.002*	21	0.005*
Mann–Whitney U (Tb vs Te)																				
Experimental	4	**0.027**	8	0.098	4	0.055	9	0.469	4	**0.014**	2	**0.012**	4	0.055	3	0.078	2	**0.023**	0	0.002*
Control	22	0.11	31	0.898	3	**0.010**	8	**0.006**	4	0.002*	10	0.084	13	0.160	18	0.057	22	0.110	12	**0.017**
Isometric Force																				
Wilcoxon Matched Pairs tests (experimental vs control)																				
Tb	39	0.114	34	0.057	39	0.115	41	0.262	42	0.166	64	0.976	56	0.605	39	0.209	35	0.067	52	0.695
Te	27	**0.018**	23	**0.017**	45	0.232	56	0.605	45	0.393	51	0.410	48	0.313	34	0.057	59	0.738	34	0.057
Mann–Whitney U (Tb vs Te)																				
Experimental	3	**0.010**	3	**0.020**	8	**0.049**	0	0.004*	7	0.074	7	**0.037**	13	0.160	3	**0.020**	12	0.131	6	0.055
Control	14	**0.027**	12	**0.017**	5	0.002*	8	**0.006**	6	0.003*	30	0.305	20	0.080	13	**0.021**	3	0.001*	26	0.191
Matching Error																				
Wilcoxon Matched Pairs tests (experimental vs control)																				
Tb	41	0.148	43	0.324	38	0.277	29	**0.026**	40	0.235	62	0.879	58	0.693	56	0.605	43	0.324	57	0.648
Te	59	0.738	40	0.345	49	0.343	48	0.456	46	0.557	51	0.809	37	0.247	40	0.131	55	0.563	60	0.784
Mann–Whitney U (Tb vs Te)																				
Experimental	22	0.625	16	0.496	28	0.57	12	0.131	15	0.426	32	0.301	19	0.432	21	0.91	27	0.652	23	0.695
Control	71	0.080	49	0.470	46	0.622	66	0.168	38	0.635	41	0.910	48	0.893	55	0.054	59	0.376	44	0.946

Results of Mann–Whitney U Tests to detect the presence of group differences (“Experimental vs Control”) and of Wilcoxon Matched Pairs Tests to inspect changes inside each group (“Tb vs Te”). Cells present p-values related to each specific direction of movement, tested in each exercise. p-values in bold are significant at p < 0.05. Asterisks (*) show which results are still significant after Bonferroni correction for multiple comparisons (p-values significant at p < 0.005)

### Isometric force

**Fig. 7:**
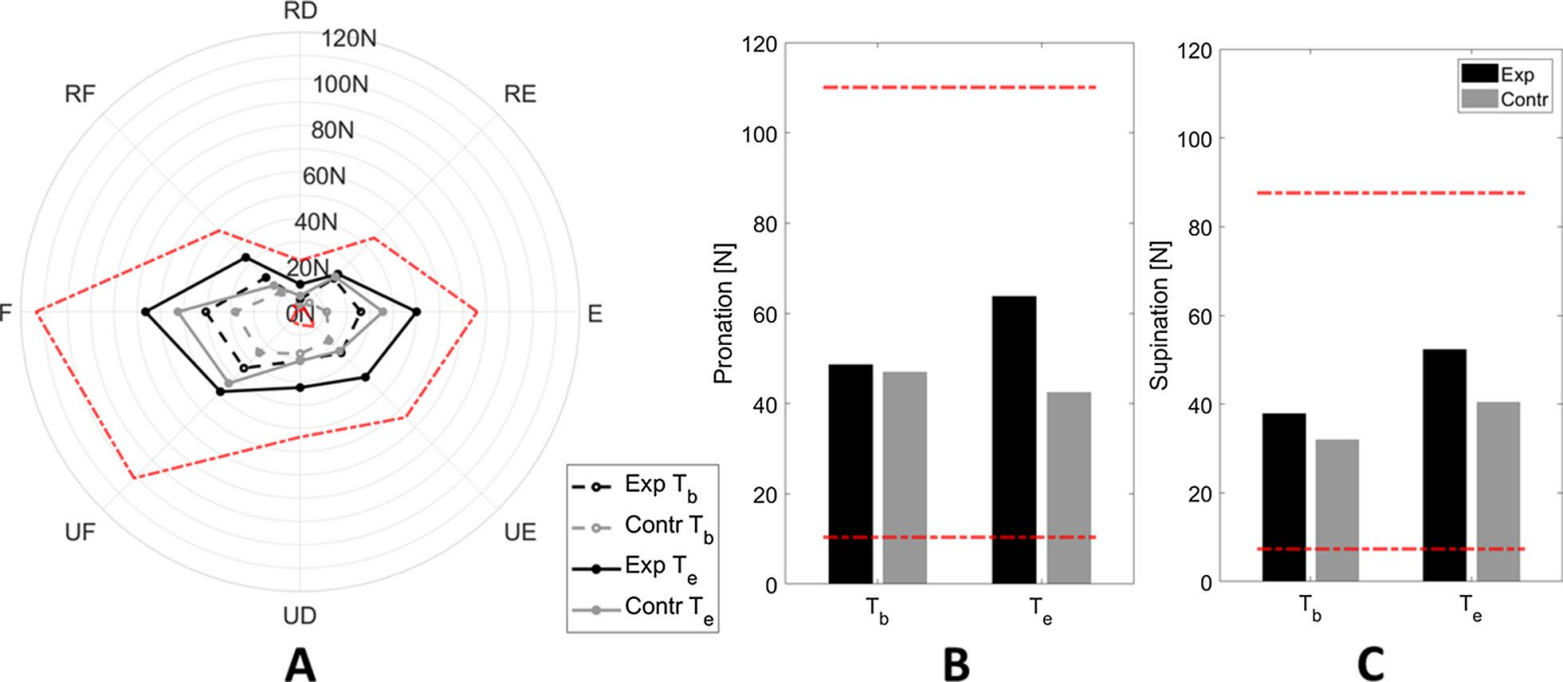
Median values of isometric force. **A** showed directions on the FE-RUD space, while **B**, **C** pronation and supination direction, respectively. Black and grey lines stay for experimental and control group, respectively. In panel A dotted and solid lines stay for T_b_ and T_e_ assessment, respectively. Red dotted lines represented data used to normalize each direction, i.e., the best and worst performance (*x*_*best*_ and* x*_*worst*_) found along that specific direction, among all the subjects and evaluations

Globally, subjects’ isometric force (*outcome*_*isoforce*_) increased in both groups. Groups presented comparable values of force at T_b_ (U = 38, p = 0.101), not only considering *outcome*_*isoforce*_, but also single directions (Table 4 and Fig. 7). After rehabilitation, both groups improved (experimental: W = 50, p = 0.020; control: W = 88, p = 0.001): this force growth was similar in the two groups and Mann–Whitney U tests showed that achieved force values were still comparable at T_e_ (U = 34, p = 0.057) (Table 3).

### Target tracking

Differently from the previous analyzed tasks, target tracking performance was evaluated only through a single outcome measure, i.e., the mean figural error in the two completed laps (see “Subjects and experimental protocol”), without any distinction about movement direction and single DoFs. This indicator metric was processed as described in Eq. 1: normalized median values are shown in Table 3 (*outcome*_*tracking*_ computed with* x*_*best*_ = 0.6°,* x*_*worst*_ = 5.9°), while median figural errors in degrees of each group at each assessment are reported in the following lines. Statistical analysis revealed that target tracking performance was comparable between groups both before (U = 57, p = 0.648; experimental: 1.7°, control: 1.7°) and after three weeks of rehabilitation (U = 36, p = 0.077; experimental: 1.2°, control: 1.5°). Although groups presented comparable final errors, only the experimental group resulted to be improved significantly from T_b_ to T_e_ (experimental: W = 47, p = 0.049; control: W = 53, p = 0.635).

### Joint position matching

Analogously to what presented for the Target Tracking outcomes, also matching errors were comparable between groups both before (U = 43, p = 0.186) and after three weeks of rehabilitation (U = 53, p = 0.483).

**Fig. 8:**
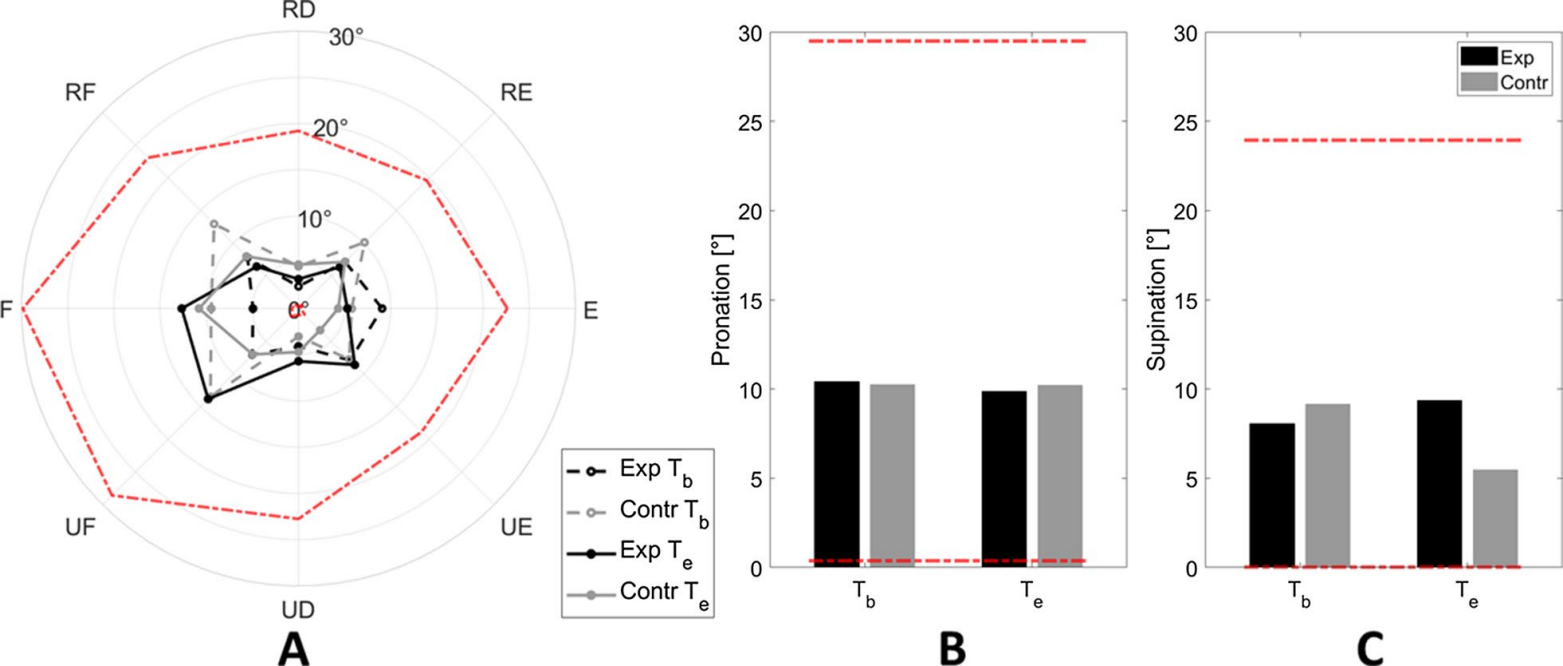
Median values of matching error. **A** showed directions on the FE-RUD space, while **B**, **C** pronation and supination direction, respectively. Black and grey lines stay for experimental and control group, respectively. In panel A dotted and solid lines stay for T_b_ and T_e_ assessment, respectively. Red dotted lines represented data used to normalize each direction, i.e., the best and worst performance (*x*_*best*_ and* x*_*worst*_) found along that specific direction, among all the subjects and evaluations

As can be guessed by Fig. 8, for the experimental group neither most single directions (Table 4) nor median *outcome*_*jpm*_ (Table 3) showed improvements in the perception of wrist position (W = 22, p = 0.625). Conversely, control subjects showed a slight improvement in their performance (W = 77, p = 0.027).

### Perceived satisfaction and acceptability

After the last robotic evaluation, subjects’ experienced robotic training and/or assessment was scored with a median value of 10 out of 10 (mean: 9.67, SE: 0.333) by the experimental group, and 9.5 (mean: 9.33, SE: 0.333) by the control group. In both groups, no important harm or adverse event was reported.

## Discussion

This randomized controlled trial tested the efficacy of a robot-based rehabilitative protocol to recover wrist functionality after traumatic injuries. The efficacy of robotic devices in the orthopedic field was investigated in a restricted number of studies [16, 17], whose main limitation can be found in the absence of a comparison with a traditional treatment. To investigate the efficacy of this novel rehabilitative approach, our protocol involved sessions of assessment pre- and post-rehabilitation, including both robot-aided measurements and evaluations through traditionally used clinical scales. The assessed measurements aimed to point out whether robot-based rehabilitation (10 experimental subjects) had an effect, in terms of presence of improvements in functionality, and whether the final performance was comparable to that achieved after a traditional rehabilitative protocol (13 control subjects). The structure of both the robot-based rehabilitative protocol and the traditional one was always chosen by the medical specialist, tailoring the treatment accordingly to the severity and the level of healing achieved by each subject. Robot-based exercises replaced traditional exercises preserving the corresponding goal, such as reducing rigidity [6] or improving stretch, muscle force or dexterity [7, 10, 42].

Clinical results revealed that the robot-based rehabilitative approach was effective and that its results were comparable to those achieved through traditional exercises. Actually, the function subscale of PRWE pointed out that both robot-aided and traditional rehabilitation led to a recover of wrist functionality and that experimental and control group were comparable both before and after rehabilitation.

The entire set of clinical measures, Jamar Test, Jebsen-Taylor Test and PRWE, showed that grip force, dexterity, pain, and functionality were comparable between groups, both pre- and post-treatment, stating the comparability between robot-based and traditional approaches. Considering long-term effects, in our study only the control group resulted to have further improved its score in the function subscale of the PRWE at the follow-up assessment. Retaining improvements beyond the period of training is a crucial goal for rehabilitation and evidences of a long-term retention after robotic rehabilitation have already been found in the neuro-rehabilitative field [43–46]. Whether and how retention of functionality is related to the employment of robotic devices for orthopedic rehabilitative treatments should be investigated further.

Robotic assessments showed that both the experimental and the control group significantly improved their performance in robot-based tasks, however, subjects that underwent robot-aided rehabilitation presented a better outcome performance respect to traditionally treated ones. It is crucial to highlight that, since we aimed to avoid speculating on results derived from a more intensive use of the device by the experimental group, tasks used for the robot-aided assessment were designed to be deeply different from those used to treat subjects. Additionally, at each assessment, all subjects had time to familiarize with the device before being tested. Despite this, subjects were also evaluated through clinical scales and their results discussed, removing any possible effect affecting the comparison, related to a more prolonged use of the device.

In details, greater improvements were found in ROM and measures of isometric forces in both groups: subjects’ rigidity decreased, allowing wider movements, and muscle force increased in the isometric task. The improvements in ROM and force generation capacity, reported for the experimental group, are in accordance with what was found for the lower limb by Deuthsch et al. [17], using a haptic interface for rehabilitation after ankle injuries.

Contrarily, improvements were not so great and clear in the Joint Position Matching and in the Tracking task. It is worth remembering that these tasks were tailored accordingly to subjects’ passive ROM. However, the influence of movement amplitude on wrist proprioceptive acuity and tracking accuracy is well known [39, 47]: presence of different ROM among subjects and between assessments, and the subsequent testing with disparate movement amplitudes, could have led to increase the variability of our results, hiding any possible change exclusively related to an improved perception of wrist position. Additionally, another limitation of our study was related to the size of the Lissajous figure considered in the Tracking task: although the size changed accordingly to a percentage of subjects’ passive ROM, the tracking task required active movements and, as shown in Fig. 5, at the first evaluation the median active ROM was visibly smaller than the passive one.

Although this study evaluated subjects’ performance through indicators widely used in robotic rehabilitation [38, 39], future studies should address the above-presented issues and, still preserving safe conditions in presence of reduced ROM, find novel tasks and outcome measures of assessment independent from movement amplitude.

Interestingly, even though our sample presented a wide variety and differently treated injuries [1], before rehabilitation groups were comparable in both outcome metrics of each robotic task and clinical measures assessed. Since inclusion criteria did not restricted participants on the basis of the wrist injury or conservative/surgery treatment they got, statistical dispersion of our results was broad: one limitation of this study was related to the high variability of our sample in terms of both sensory and motor performance, that led subjects to be impaired along specific and different directions of movement.

Overall, robotic and clinical results agreed in stating that robot-based rehabilitation was effective and comparable with a traditional protocol. Future studies should address a deeper knowledge about the correlation between clinical and robotic outcomes and whether different robot-based metrics [22, 48] could assess other components influencing wrist recovery of functionality in post-traumatic subjects. Although the correlation analysis was not a primary objective of this study, we performed some potentially interesting correlations on our sample of clinical and robotic measures. From preliminary Pearson correlation analyses, we obtained a significant correlation between isometric force (*outcome*_*isoforce*_) and grip force measured in the Jamar test (r = − 0.36, p = 0.013), where the negative correlation results from the processing and computation of outcome_isoforce_, with lower values indicating higher forces exerted (see “Data Processing”). Additionally, the *Robotic Assessment Index* resulted significantly correlated with the function subscale of the PRWE (r = 0.326, p = 0.033), with lower values of both metrics pointing out improved wrist functionality. Even though these analyses showed a relation between novel robotic outcomes and largely employed clinical measures, more interesting and robust results could be obtained in future studies involving larger samples.

Given our results, it is evident that research should particularly focus on developing both somatosensory robot-aided assessments and trainings [19–21], suitable for orthopedic subjects. However, although the proposed robot-aided training employed exercises designed for neuro-rehabilitative purposes [38, 49, 50], these resulted well-tolerated by post-traumatic subjects. Similarly to what other studies reported using a different robotic device for orthopedic rehabilitation [16], our sample of patients resulted satisfied and well-accepted the device: acceptability was rated with excellent scores, even higher in the experimental group, whose both rehabilitation and assessment were centered on its employment.

The potential of a systematic use of robotic devices in orthopedics is twofold: besides increasing accuracy and repeatability in the assessment of functionality, robot-based rehabilitation could be maximally exploited tailoring rehabilitative protocols real-time to target to subject’s specific functional deficits and promote his/her voluntary participation [51], minimizing time duration and therapists’ effort during rehabilitation.

## Conclusions

This work aimed to test a robot-based rehabilitative approach on orthopedic subjects. Our results showed that the robot-aided protocol of treatment was effective and comparable to the traditional one. Despite our sample presented a wide variety of wrist injuries, subjects’ wrist functionality was comparable before the treatment considering both robotic evaluations and assessed clinical measures and scales. After the three-week long rehabilitation, clinical results showed that groups did not differ in terms of functionality, pain, grip force and dexterity. The robotic assessment showed that the experimental group presented greater improvements than the control group, particularly in terms of reduced tissue rigidity and increased muscle force. This work can be considered as a starting point for introducing the use of robotic devices in the orthopedic field, where a systematic use of these devices could assist therapists’ work and increase accuracy in tailoring treatments to target specific injury-related issues.

## Data Availability

The datasets used and/or analyzed during the current study are available from the corresponding author on reasonable request.
